# Research on Denoising Methods for Laser Doppler Blood Flow Signals Based on Time-Domain Noise Perception and DWT

**DOI:** 10.3390/s26051500

**Published:** 2026-02-27

**Authors:** Quanxin Sun, Jie Duan, Hui Guo, Aoyan Guo

**Affiliations:** 1 School of Optoelectronic Engineering, Changchun University of Science and Technology, Changchun 130013, China; 2023100361@mails.cust.edu.cn; 2 CRRC Changchun Railway Vehicles Co., Ltd., Changchun 130062, China; gh95272026@163.com (H.G.); 2024100402@mails.cust.edu.cn (A.G.)

**Keywords:** discrete wavelet transform, temporal noise perception, laser Doppler, adaptive denoising

## Abstract

Addressing the challenges of composite noise (speckle noise, thermal noise, and random pulse interference) and non-stationarity in laser Doppler flow (LDF) signal processing, as well as the technical limitation of traditional threshold methods in balancing noise suppression and signal fidelity, this study proposes an adaptive denoising algorithm integrating temporal noise perception and discrete wavelet transform (DWT). A composite noise model is first established to characterize the interference. The signal undergoes a five-level DWT decomposition, where a local energy detection mechanism distinguishes signal-dominant from noise-dominant regions. An SNR-driven dynamic thresholding strategy is generated by combining inter-layer adaptive allocation with coefficient-level local weighting, followed by processing with an improved smoothing function to effectively suppress reconstruction artifacts. Simulations at a 1 dB input signal-to-noise ratio (SNR) yielded a 15.45 dB output SNR and a 0.05634 root mean square error (RMSE), outperforming traditional wavelet methods and modern benchmarks such as local variance and variational mode decomposition (VMD). Applied to a practical signal from an isolated vascular phantom with an initial SNR of −1.04 dB, the algorithm achieved a 13.86 dB output SNR and a 0.00258 RMSE. Results confirm the algorithm’s effectiveness for high-fidelity waveform capture in complex noise environments, offering a robust solution for vascular hemodynamic monitoring

## 1. Introduction

Accurate and non-invasive measurement of blood flow velocity and volume is essential for applications such as vascular hemodynamic monitoring [1], tissue perfusion assessment [2], and diagnosis of chronic limb-threatening ischemia [3]. Currently, various optical techniques have been developed, each with distinct emphases. For instance, diffuse correlation spectroscopy (DCS) and diffuse speckle contrast analysis (DSCA) techniques, based on photon diffusion theory, effectively assess deep tissue blood perfusion at depths exceeding 1 mm. The recently developed diffuse speckle pulsatile flowmetry (DSPF) technology further enhances the sampling rate to over 300 Hz using multi-mode detection fibers, enabling fine capture of tissue perfusion dynamics at depths of approximately 6 mm; the tissue perfusion index (TPI_DSPF) defined based on this technology demonstrates excellent clinical value in diagnosing peripheral arterial disease (PAD) in diabetic feet [4,5]. The core advantage of these “diffuse optical techniques” lies in assessing the comprehensive perfusion level of deep tissue microcirculation. However, their measurement principle primarily reflects the statistical average state (i.e., perfusion) of numerous randomly moving scatterers within the detection volume. In contrast, when the measurement objective requires direct, high-fidelity capture of high-frequency, transient velocity waveforms within superficial and relatively isolated vascular structures (or in vitro tubing), Laser Doppler Flowmetry (LDF) technology is often better suited due to its direct velocity sensitivity derived from the Doppler shift principle.

LDF enables non-contact measurement of blood flow dynamics by detecting the Doppler shift in scattered light. The signals obtained, however, present distinct and challenging characteristics for processing: they are modulated by cardiac pulsation and respiratory rhythm, leading to significant local energy fluctuations in the time domain and pronounced time-varying non-stationarity [6]; speckle noise, arising from the random distribution of red blood cells and laser coherence, has a spectrum that highly overlaps with the effective blood flow signal, making separation difficult with conventional filtering methods [7]; and the signals often contain transient peaks that are easily masked by noise or misidentified [8]. Additionally, practical measurement systems introduce thermal noise and random electromagnetic interference, creating a challenging composite noise environment. These intertwined characteristics—non-stationarity, composite noise, and transient features—make achieving a balance between noise suppression and signal fidelity a critical yet difficult challenge in LDF signal processing.

The inherent complexity and stochastic nature of light scattering in real biological tissues make it difficult to establish a precise ground truth for evaluating signal processing algorithms. To rigorously validate the core denoising capability addressing the above challenges, this study first adopts a controlled and deterministic physical model—an isolated vessel model represented by an in vitro simulated tubing system. This simplified model provides a well-defined scenario where the flow velocity and the detected Doppler signal follow a deterministic physical relationship (approximating single scattering dominance). It allows for the generation of a known reference signal and the controlled introduction of composite noise, thereby enabling a clear and quantitative performance benchmark for the proposed algorithm. The validation in this foundational model is a crucial step prior to future applications in more complex physiological environments.

The wavelet transform, due to its time–frequency localization capability, has been widely applied to LDF signal processing [9]. However, many denoising methods have been proposed for other types of laser Doppler signals, such as velocimetry, vibration measurement, and anemometry, and their direct application to LDF signals suffers from insufficient adaptation. For instance, Liu et al. [10] denoised laser Doppler velocimetry signals by constructing an adaptive wavelet threshold function, effectively improving the signal-to-noise ratio (SNR) in steady-state mechanical motion measurement. However, its threshold strategy is primarily designed for stationary signals and proves inadequate in handling the coupled interference of speckle noise and time-varying physiological pulsations when applied to blood flow signals. Tan et al. [11], targeting laser Doppler vibration signals, introduced a scale factor to optimize the wavelet threshold, significantly suppressing high-frequency noise induced by mechanical vibrations. Yet, the noise suppression mechanism of this method is tailored for mechanical vibrations, differing from the composite noise characteristics in blood flow signals originating from vascular wall elastic fluctuations and tissue scattering. Zhang et al. [12] employed a variational mode decomposition method optimized by the honey badger algorithm to process signals from coherent Doppler wind lidar, enhancing the suppression of atmospheric turbulence noise under low SNR conditions. However, the time–frequency distributions of wind field noise and blood flow speckle noise differ significantly. At low SNR, mode mixing is likely to occur, making it difficult to distinguish noise from high-frequency physiological details in blood flow signals. Chen et al. [13] achieved high-precision frequency estimation for laser Doppler velocimetry signals by constructing a hybrid convolution window to suppress spectral leakage. However, this method relies on the global stationarity assumption of the Fourier transform, limiting its ability to capture the time-domain non-stationary structure and transient features of blood flow signals. Furthermore, traditional hard/soft threshold functions suffer from pseudo-Gibbs oscillations or constant bias [14], further affecting the accurate preservation of transient peaks and waveform fidelity in blood flow signals.

To address the above issues, this paper proposes an adaptive denoising algorithm that integrates temporal noise perception and discrete wavelet transform (DWT). Applied to an isolated vascular flow velocity measurement experiment (with potential applicability to clinical scenarios such as intraoperative graft flow monitoring and extracorporeal circulation velocity assessment), this algorithm effectively resolves the waveform distortion problem in laser Doppler blood flow signals caused by composite noise and non-stationarity, successfully overcoming the technical bottleneck of traditional methods in balancing noise suppression and signal fidelity. This method provides a robust technical solution for high-precision blood flow monitoring in controlled vascular environments and offers theoretical support for the development of related blood flow detection devices. Its core denoising mechanism is capable of addressing the universal challenge of composite noise in more complex biological environments, thereby laying a methodological foundation for the future extension of this approach to clinical applications such as transcutaneous microvascular diagnostics.

## 2. Signal Model

### 2.1. Laser Doppler Blood Flow Signal

**Figure 1:**
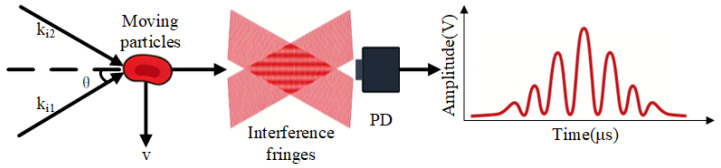
Schematic of dual-beam differential Laser Doppler Flowmetry principle.

The principle of Laser Doppler Flowmetry (LDF) relies on the Doppler shift of light scattered by moving red blood cells. In this study, a dual-beam differential configuration is adopted to enhance signal stability. As illustrated in Figure 1, two coherent laser beams with wave vectors $k_{i1}$ and $k_{i2}$ intersect at the detection zone, illuminating a red blood cell moving with a velocity vector v.

According to photon scattering theory, the Doppler frequency shift introduced by each beam is determined by the dot product of the scattering vector and the velocity vector. Due to the square-law detection characteristic of the photodetector (PD), the scattered light from the two beams interferes (mixes) on the detector surface. This heterodyne mixing generates a beat frequency in the output electrical current, which corresponds to the differential Doppler shift $f_{D}$ (scalar, unit: Hz):

(1) $$f_{D}=\frac{1}{2\pi }\left(k_{i2}-k_{i1}\right)\cdot v$$

where v represents the velocity vector of the red blood cell (unit: m/s). For the specific optical geometry where the half-angle between the two beams is θ, this differential shift can be simplified to the scalar form [15]:

(2) $$f_{D}=\frac{2|v|}{\lambda }\sin\theta$$

where |v| is the flow speed; λ is the laser wavelength (unit: nm); and θ is the half-angle between the two beams. Equation (2) serves as the basis for calculating the effective beat frequency in our model, linking the electrical signal frequency directly to the blood flow velocity.

In actual blood flow, red blood cells are distributed as discrete particles passing through the detection volume. The scattering signal from a single red blood cell can be modeled as a sinusoidal wave modulated by a Gaussian envelope, expressed as Equation (3) [16]:

(3) $$s_{i}\left(t\right)=A_{i}\exp\left(-\frac{{\left(t-t_{i}\right)}^{2}}{2\sigma^{2}}\right)\sin\left(2\pi f_{D}\left(t-t_{i}\right)\right)$$

where $A_{i}$ (unit: V) is the scattering amplitude; $t_{i}$ (unit: s) is the transit time; and σ (unit: s) is the standard deviation of the Gaussian envelope, related to the beam waist and the cell’s trajectory.

Considering the parabolic velocity profile within a vessel and the inherent non-stationary nature of blood flow, red blood cells traveling at different velocities exhibit time-varying, broad-spectrum scattering characteristics. The detector output is the superposition of these multiple Doppler-shifted components. To simulate realistic vascular flow characteristics in an isolated vessel or phantom, the model is extended to:

(4) $$s\left(t\right)=\sum_{i=1}^{M}a_{i}\left(t\right)\sin\left(2\pi {f_{D}}^{\left(i\right)}t+\phi_{i}\left(t\right)\right)$$

where *M* is the total number of scatterers contributing to the signal; $f_{D}^{\left(i\right)}$ (unit: Hz) denotes the Doppler frequency shift corresponding to the *i*-th scatterer component; $a_{i}\left(t\right)$ (unit: V) is the time-varying amplitude; and $\phi_{i}\left(t\right)$ (unit: rad) is a random phase term.

Modeling of Non-Stationarity and Spectral Characteristics: The time-varying amplitude $a_{i}\left(t\right)$ introduced in Equation (4) aims to simulate the non-stationary physiological characteristics of blood flow, specifically including: periodic blood flow fluctuations modulated by cardiac pulsation or vasomotion; and random variations in scatterer density within the detection volume. The phase term $\phi_{i}\left(t\right)$ incorporates random phase perturbations to account for the effects of red blood cells randomly entering and exiting the detection zone, as well as cell tumbling motion. Furthermore, to mimic the characteristics of real blood flow power spectra (where energy density typically decays monotonically with increasing frequency), the amplitudes of different Doppler components are set to decrease with increasing frequency shift.

Simulation Parameter Settings: To establish an ideal simulation condition, the parameters are configured as follows: laser wavelength λ=632.8nm, and the half-angle between the two beams $\theta =30^{\circ }$. To cover the typical range of blood flow velocities, five different flow speeds were selected: v=[12.7,63.3,113.9,164.5,202.5]mm/s. According to Equation (2), the corresponding Doppler shift components $f_{D}^{\left(i\right)}$ are calculated as [20,100,180,260,320]kHz. The corresponding initial time-varying amplitudes are set as $a_{i}=\left[1.0,0.6,0.4,0.3,0.2\right]$ to reflect the aforementioned spectral decay characteristic. This setting not only covers conventional microcirculation flow velocities (approximately 20 kHz) but also extends to high-velocity blood flow scenarios, thereby comprehensively evaluating the algorithm’s performance under various conditions.

To align with the high-speed signal acquisition hardware required for future embedded implementation, and to utilize oversampling gain for reducing the quantization noise floor and improving the signal-to-noise ratio (SNR), the sampling frequency was set to 125 MHz.

**Figure 2:**
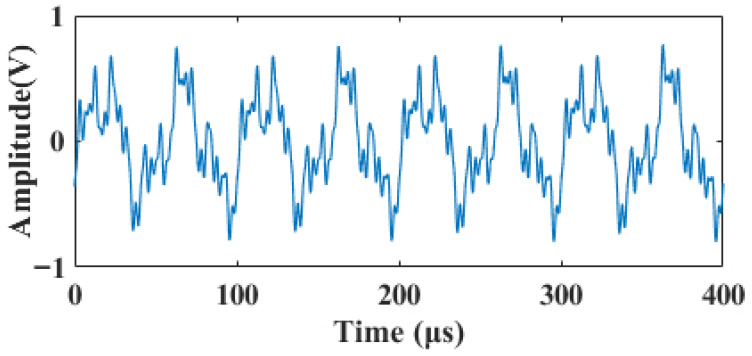
Ideal laser Doppler blood flow simulation signal.

The generated ideal laser Doppler blood flow signal waveform is shown in Figure 2.

### 2.2. Composite Noise

**Figure 3:**
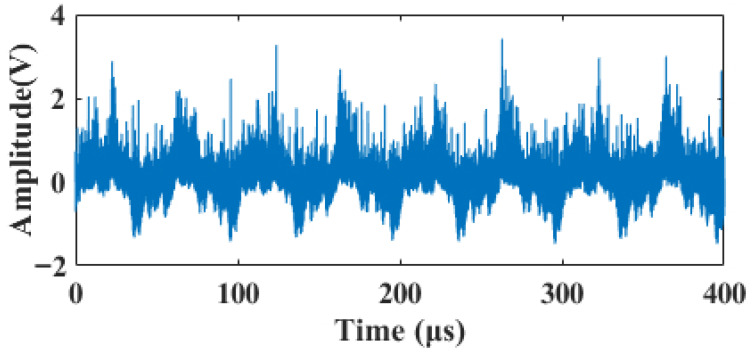
Noise laser Doppler blood flow simulation signal.

The actually acquired laser Doppler blood flow signal is susceptible to interference from multiple types of noise, primarily including speckle noise [17], thermal noise [15], and random pulse interference [18]. A composite noise model is adopted using additive superposition. Specifically, speckle noise is generated using a Gamma distribution, incorporating low-frequency time-varying modulation and nonlinear coupling with the signal amplitude to simulate the time-varying non-Gaussian disturbances caused by scattering. Thermal noise consists of white noise, 1/f (pink) noise, random burst noise, and high-frequency bandpass components, characterizing the broadband and high-frequency thermal noise properties of the detector. Pulse interference is modeled using asymmetric pulses with random positions, amplitudes, and widths, superimposed with decaying ringing to simulate transient electromagnetic/electronic interference. To ensure comparability across different experimental conditions, the total noise is uniformly adjusted via energy scaling according to the target signal-to-noise ratio (SNR), so that the generated noisy signal meets the specified SNR. The noisy signal with an SNR of 1 dB is shown in Figure 3.

## 3. Denoising Algorithm Design

**Figure 4:**
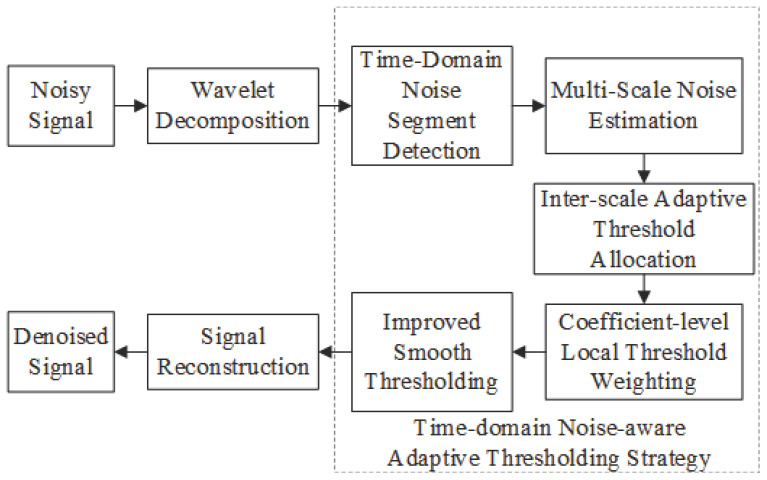
Flow of time-domain noise-aware and DWT adaptive denoising algorithm.

The composite noise in laser Doppler blood flow signals exhibits significant heterogeneous distribution characteristics in the frequency domain, which limits the performance of conventional fixed-threshold methods due to their lack of adaptability when dealing with such composite interference. To address the aforementioned issues, the overall workflow of the time-domain noise-aware and DWT-based denoising algorithm proposed in this paper is illustrated in Figure 4.

### 3.1. Wavelet Decomposition

This paper employs the Mallat fast algorithm [19] for discrete wavelet decomposition, as shown in Equation (5).

(5) $$\left\{\begin{array}{c} a_{j-1}\left[k\right]=\sum_{n}h\left[n-2k\right]a_{j}\left[n\right]\\ d_{j-1}\left[k\right]=\sum_{n}g\left[n-2k\right]a_{j}\left[n\right] \end{array}\right.$$

where $a_{j}$ represents the approximation coefficients at the *j*-th level; $d_{j-1}$ denotes the detail coefficients at the (j−1)-th level; k=1,2,3,…,N−1 is the coefficient index at the current level; *h* is the low-pass filter; *g* is the high-pass filter; and *n* represents the number of discrete sampling points, corresponding to the length of the signal sequence at the previous level.

The selection of the decomposition level and the matching between the wavelet basis function and the signal characteristics are critical to wavelet decomposition. This paper adopts a 5-level wavelet decomposition. Under a sampling rate of 125 MHz, the corresponding frequency bands for each level are: D1 (31.25–62.5 MHz), D2 (15.625–31.25 MHz), D3 (7.8125–15.625 MHz), D4 (3.90625–7.8125 MHz), D5 (1.953125–3.90625 MHz), and A5 (0–1.953125 MHz). The primary frequency components of the blood flow signal (20–320 kHz) are concentrated in the A5 level, while high-frequency noise is distributed across levels D1 to D4. This decomposition depth effectively separates the signal from the noise.

Three wavelet bases commonly used in laser Doppler signal processing, db8, sym8, and coif5, were selected. The ideal signal shown in Figure 2 was decomposed into 5 levels according to Equation (5) and then reconstructed using Equation (20). The reconstruction performance was evaluated using Mean Squared Error (MSE) and Mean Phase Distortion (rad) as metrics. The results presented in Table 1 show that sym8 achieves the minimum values in both metrics. Therefore, sym8 is chosen as the wavelet basis function for this study.

### 3.2. Time-Domain Noise-Aware Adaptive Thresholding Strategy

The core design of this strategy lies in capturing the local energy fluctuation characteristics of the signal from the time-domain perspective, thereby enabling accurate discrimination between signal-dominant segments and noise-dominant segments. Based on this discrimination, the threshold weight is dynamically regulated: it is adaptively increased in noise-dominant intervals to enhance noise suppression effectiveness, while appropriately reduced in signal-dominant intervals to preserve high-frequency signal details. Ultimately, this strategy establishes a dynamically optimal balance between signal fidelity and noise suppression capability.

#### 3.2.1. Time-Domain Noise Segment Detection

Following wavelet decomposition, noise segment detection is performed on the original time-domain signal f(n). The local energy $E_{\mathrm{local}}$ is calculated within a sliding window of length *L* and then normalized to obtain $E_{\mathrm{norm}}$:

(6) $$E_{\mathrm{local}}\left(n\right)=\mathrm{MovMean}\left(f^{2}\left(n\right),L\right)$$

(7) $$E_{\mathrm{norm}}\left(n\right)=\frac{E_{\mathrm{local}}\left(n\right)}{\max(E_{\mathrm{local}})}$$

where MovMean(·,L) denotes a moving average filter with a window length of *L*, set to 500 in this paper, corresponding to a 4 μs temporal window at the 125 MHz sampling rate. This length is chosen to be shorter than the typical duration of transient flow events while providing sufficient statistical stability for local energy estimation; and *n* is the sample index. Based on the statistical distribution characteristics of the signal energy, an adaptive energy threshold is constructed using the median and standard deviation (std):

(8) $$T_{\mathrm{energy}}=\mathrm{median}\left(E_{\mathrm{norm}}\left(n\right)\right)-1.5\times \mathrm{std}\left(E_{\mathrm{norm}}\left(n\right)\right)$$

where the coefficient 1.5 is determined based on the 3σ criterion of the normal distribution to identify low-energy regions with 99.7% confidence. The time-domain noise segment index $M_{\mathrm{noise}}$ is generated as:

(9) $$M_{\mathrm{noise}}=\left\{n|E_{\mathrm{norm}}\left(n\right)<T_{\mathrm{energy}}\right\}$$

#### 3.2.2. Multi-Scale Noise Estimation

Due to the multi-resolution nature of wavelet decomposition, the sampling rate of the coefficients at the *j*-th level is $1/2^{j}$ of the original signal. Therefore, the index $M_{\mathrm{noise}}$ is downsampled by a factor of $2^{j}$ to map it to the corresponding decomposition level, extracting the noise component $d_{j}^{noise}$ from the wavelet coefficients at each level. The noise standard deviation $\sigma_{j}$ for the *j*-th level is estimated using the median absolute deviation (MAD), and the global noise standard deviation $\sigma_{\mathrm{global}}$ is calculated as follows:

(10) $$\sigma_{j}=\mathrm{median}(|d_{j}^{\mathrm{noise}}\left|\right)/0.6745$$

(11) $$\sigma_{\mathrm{global}}=\frac{1}{J}\sum_{j=1}^{J}\sigma_{j}$$

where *J* is the total number of wavelet decomposition levels (here J=5), and the coefficient 0.6745 is the conversion factor from MAD to standard deviation under a standard normal distribution. This process utilizes noise segments for statistics, effectively avoiding overestimation of noise levels caused by strong signals.

#### 3.2.3. Inter-Scale Adaptive Threshold Allocation

Considering the distribution differences of signals and noise across different decomposition frequency bands, an inter-scale base threshold $T_{j}$ is constructed to achieve frequency-band-specific processing:

(12) $$T_{j}=\sigma_{\mathrm{global}}+\gamma \cdot \sigma_{j}$$

where γ is an inter-scale adjustment factor. To prevent extreme cases of highly uneven noise distribution, its value is limited to the range [2,4.5], the lower bound of 2 aligns with the 2σ rule for Gaussian noise, while the upper bound of 4.5 approximates the universal threshold $\sigma \sqrt{2\ln N}$ for a typical signal length *N*, providing a theoretically justified limit. And dynamically determined by the coefficient of variation (CV) of the noise distribution across levels, i.e., the ratio of the standard deviation to the mean:

(13) $$CV_{\sigma }=\mathrm{std}\left(\sigma_{j}\right)/\mathrm{mean}\left(\sigma_{j}\right)$$

(14) $$\gamma =2+5\cdot \min(CV_{\sigma },0.5)$$

when $CV_{\sigma }$ is high, indicating uneven noise distribution across frequency bands, γ automatically increases to enhance threshold differences between bands. Conversely, thresholds across levels tend to converge, achieving adaptive matching to different noise characteristics.

#### 3.2.4. Coefficient-Level Local Threshold Weighting

To address the issue of weak signal detail loss caused by fixed thresholds, a point-by-point adaptive weighting mechanism is introduced. First, the local variance LocalVar(f) of the time-domain signal within a sliding window is calculated. The coefficient of variation is used as an indicator of non-stationarity in the time-domain signal to define a local sensitivity factor β. To prevent excessive modulation that could distort the signal waveform, β is constrained to the range [1,3]. This range establishes a safe dynamic range for threshold adjustment, allowing enhanced detail preservation in non-stationary regions (β>1) while capping the maximum threshold amplification to a factor of 3 to avoid introducing artifacts:

(15) $$\beta =1+2\cdot \min\left(\frac{\mathrm{std}(\mathrm{LocalVar}(f))}{\mathrm{mean}(\mathrm{LocalVar}(f))},1\right)$$

For non-stationary signals with intense fluctuations, β automatically increases to enhance detail preservation. In the wavelet domain of the *j*-th level, the normalized local energy of the coefficients is calculated to construct a dynamic adjustment factor for pointwise correction of the base threshold:

(16) $$T\left(i\right)=T_{j}\cdot \left[1+\beta \left(1-\frac{\mathrm{MovMean}(d_{j}^{2}\left(i\right),W)}{\max(\mathrm{MovMean}\left(d_{j}^{2}\left(i\right)\right))}\right)\right]$$

where *i* is the position index of the coefficient in the current decomposition level, *W* is the length of the sliding window in the wavelet domain, set to $\max(5,L_{j}/20)$, and $L_{j}$ denotes the total number of wavelet coefficients at level *j*. In high-energy, signal-dominant regions, the threshold is automatically lowered to protect transient features; in noise-dominant regions, the threshold adaptively increases to enhance suppression. Through this pointwise adjustment strategy, the threshold achieves position-dependent dynamic regulation based on the local energy characteristics of the wavelet coefficients.

#### 3.2.5. Improved Smooth Thresholding

**Figure 5:**
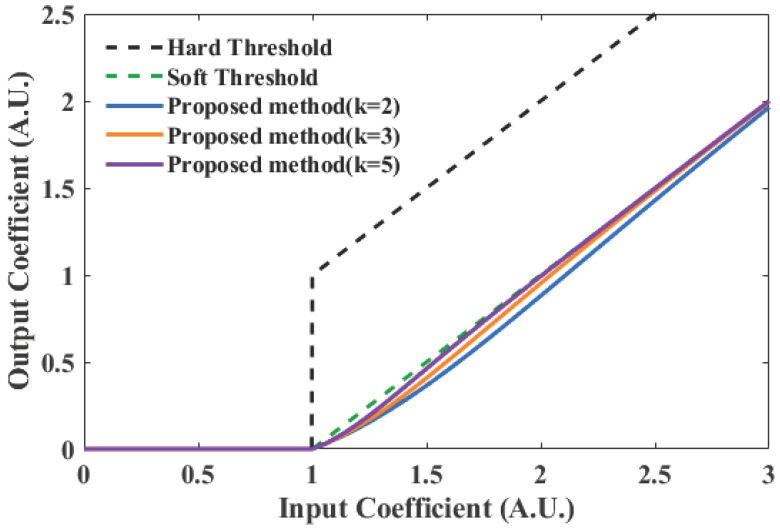
Threshold function.

This paper proposes an improved smooth thresholding function, incorporating the pointwise threshold T(i) as follows:

(17) $${\tilde{d}}_{j}\left(i\right)=\left\{\begin{array}{cc} 0 & ,|d_{j}\left(i\right)|<T\left(i\right)\\ \mathrm{sgn}\left(d_{j}\left(i\right)\right)\cdot \left(|d_{j}\left(i\right)|-\frac{T(i)}{1+\exp\left(-k\cdot (|d_{j}\left(i\right)|-T\left(i\right))\right)}\right) & ,|d_{j}\left(i\right)|\geq T\left(i\right) \end{array}\right.$$

where ${\tilde{d}}_{j}\left(i\right)$ denotes the denoised wavelet coefficient; sgn(·) is the sign function; and *k* is the smoothing control parameter, with a value range of [2,10], determined based on the estimated global signal-to-noise ratio ($SNR_{\mathrm{ext}}$) of the input signal, confined to the range based on the functional behavior of the sigmoid. Values below 2 make the transition too gradual, impairing denoising, while values above 10 cause the function to converge to a hard threshold, negating the benefits of smoothness:

(18) $$SNR_{\mathrm{ext}}=10\log_{10}\left(\frac{\mathrm{Var}(f)}{\sigma_{\mathrm{global}}^{2}}\right)$$

(19) $$k=2+3e^{-SNR_{\mathrm{ext}}/10}$$

where Var(f) represents the variance of the signal *f*. At low SNR, *k* increases, making the function approximate hard thresholding to enhance noise removal. At high SNR, *k* decreases, causing the function to tend towards linearity to preserve signal details. The function ensures continuity through a smooth transition near the threshold, while tending towards linearity away from the threshold region to overcome the constant bias inherent in soft thresholding. This achieves adaptive denoising strength according to signal quality. The first quadrant of the threshold function is illustrated in Figure 5. While sigmoid-based smoothing is known, the proposed function is primarily distinguished by its SNR-driven adaptability. The shaping parameter *k* is not fixed but dynamically mapped from the estimated global SNR, enabling automatic morphing between near-hard thresholding and near-linear scaling. This core design eliminates manual parameter tuning and ensures optimal performance across varying noise levels, which is a key advancement over static smooth functions commonly found in the literature.

### 3.3. Signal Reconstruction

#### 3.3.1. Reconstruction Algorithm

The signal reconstruction process recovers the time-domain signal through inverse operations that progressively fuse the wavelet coefficients. The Mallat pyramid reconstruction algorithm [19] is employed, as shown in Equation (20):

(20) $$\left\{\begin{array}{c} a_{j}\left[n\right]=\sum_{k}h\left[n-2k\right]{\tilde{a}}_{j-1}\left[k\right]+\sum_{k}g\left[n-2k\right]{\tilde{d}}_{j-1}\left[k\right]\\ \;f_{\mathrm{denoised}}=a_{0}\left[n\right] \end{array}\right.$$

where *h* and *g* are the reconstruction low-pass and high-pass filters, respectively; ${\tilde{a}}_{j-1}\left[k\right]$ represents the thresholded approximation coefficients at the (j−1)-th level; ${\tilde{d}}_{j-1}\left[k\right]$ represents the thresholded detail coefficients at the (j−1)-th level; *n* denotes the discrete sampling points with n=1,2,…,N; and $f_{\mathrm{denoised}}$ is the final output of the denoised time-domain signal.

#### 3.3.2. Quantitative Evaluation of Reconstructed Signal

To quantitatively evaluate the algorithm’s performance, the signal-to-noise ratio (SNR) and root mean square error (RMSE) are adopted as the core metrics:

1. The signal-to-noise ratio is given by Equation (21):
  (21) $$\mathrm{SNR}=10\cdot \log_{10}\left(\frac{P_{\mathrm{signal}}}{P_{\mathrm{noise}}}\right)$$
  where $P_{\mathrm{signal}}$ and $P_{\mathrm{noise}}$ represent the power of the clean signal and the noise component, respectively. A higher SNR value indicates better signal quality.
2. The root mean square error is given by Equation (22):
  (22) $$\mathrm{RMSE}=\sqrt{\frac{1}{N}\sum_{n=1}^{N}{\left[f_{\mathrm{denoised}}\left(n\right)-f_{\mathrm{ideal}}\left(n\right)\right]}^{2}}$$
  where $f_{\mathrm{ideal}}\left(n\right)$ is the ideal signal waveform; $f_{\mathrm{denoised}}\left(n\right)$ is the denoised signal waveform; and *N* is the number of sampling points. RMSE measures the average deviation between the denoised signal and the ideal signal, with a smaller value indicating higher reconstruction accuracy.

### 3.4. Simulation Experiment

**Figure 6:**
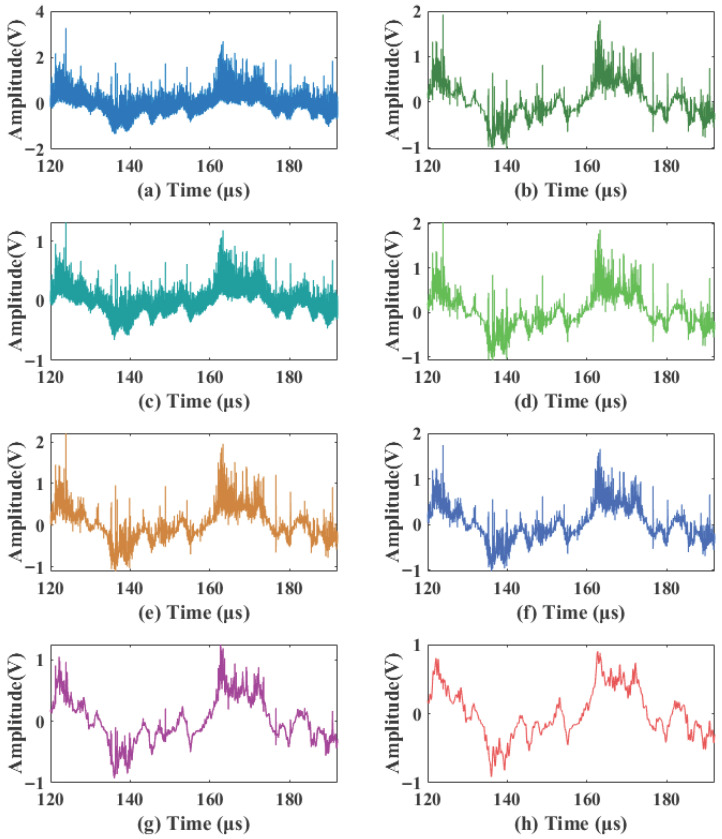
Simulation signal details comparison. (**a**) Noisy laser Doppler blood flow signal. (**b**) Local variance; (**c**) VMD; (**d**) BayesShrink; (**e**) SURE; (**f**) Improved Threshold 1; (**g**) Improved Threshold 2; (**h**) Proposed Method.

The noisy signal shown in Figure 3 was processed by the proposed algorithm and reconstructed via Equation (20). To comprehensively evaluate the performance of the algorithm under identical noise conditions, the proposed method was compared with the following algorithms: the traditional adaptive wavelet thresholding algorithms BayesShrink and SURE, two additional representative denoising methods (local variance and variational mode decomposition, VMD), the improved thresholding method 1 (Improved Threshold 1) from Ref. [20], and the improved thresholding method 2 (Improved Threshold 2) from Ref. [21]. The selection of these two improved thresholding methods as comparative baselines is clearly justified, as their core advantages are highly consistent with the key challenges of this study: Ref. [20] designs a continuous thresholding function specifically for non-stationary signal denoising, which can well match the time-varying characteristics of laser Doppler blood flow signals; Ref. [21] adopts a two-parameter thresholding function to specifically address the denoising of weak physiological signals, in line with the core requirement of this study to preserve faint transient details of blood flow signals in strong noise environments. A detailed comparison of the local waveform details of the signals denoised by each method is presented in Figure 6, and the quantitative evaluation metrics, which are the average values of six independent tests, are summarized in Table 2.

The comparison of local waveform details in Figure 6 shows that conventional denoising methods, including local variance (b), VMD (c), BayesShrink (d), and SURE (e), exhibit limited effectiveness in suppressing composite noise. Noticeable random fluctuations and speckle residues remain in their denoised waveforms; in particular, VMD (c) suffers from significant modal aliasing under strong noise conditions, leading to distorted baseline morphology. Although Improved Threshold 1 (f) reduces some noise, significant high-frequency residuals persist. Improved Threshold 2 (g) significantly attenuates speckle noise, but some thermal noise residue remains. In contrast, the proposed method (h) achieves superior noise suppression while better preserving the transient peaks and baseline morphology of the signal, demonstrating an optimal balance between noise suppression and signal fidelity.

The quantitative results in Table 2 further support this analysis. VMD and SURE yield the lowest performance, with output SNRs of 4.20 dB and 8.76 dB, respectively, indicating their difficulty in effectively distinguishing between the overlapping spectra of composite noise and the useful LDF signal. Local variance and BayesShrink achieve slightly better results, with SNRs of 9.20 dB and 9.16 dB. However, their overall performance remains significantly lower than that of the proposed method (SNR = 15.45 dB, RMSE = 0.05634), with SNR gaps exceeding 6.25 dB compared to these four baseline methods. Improved Threshold 1 increases the SNR to 10.34 dB, but its RMSE remains at 0.10145 (approximately 1.80 times that of the proposed method), suggesting insufficient adaptation to the multi-scale distribution of composite noise. The performance of Improved Threshold 2 shows notable improvement (SNR = 14.35 dB, RMSE = 0.06391), yet it is still 1.10 dB lower in SNR and approximately 13.4% higher in RMSE compared to the proposed method.

Overall, the proposed method achieves the best results in both SNR and RMSE metrics. It yields approximately 7.7% higher SNR and 11.8% lower RMSE relative to the second-best method (Improved Threshold 2). Compared to BayesShrink, the proposed algorithm improves SNR by approximately 68.7% and reduces RMSE by approximately 55.3%. These results validate the effectiveness of the proposed time-domain noise-aware and multi-scale adaptive thresholding strategy in suppressing complex composite noise while preserving critical physiological signal details.

**Figure 7:**
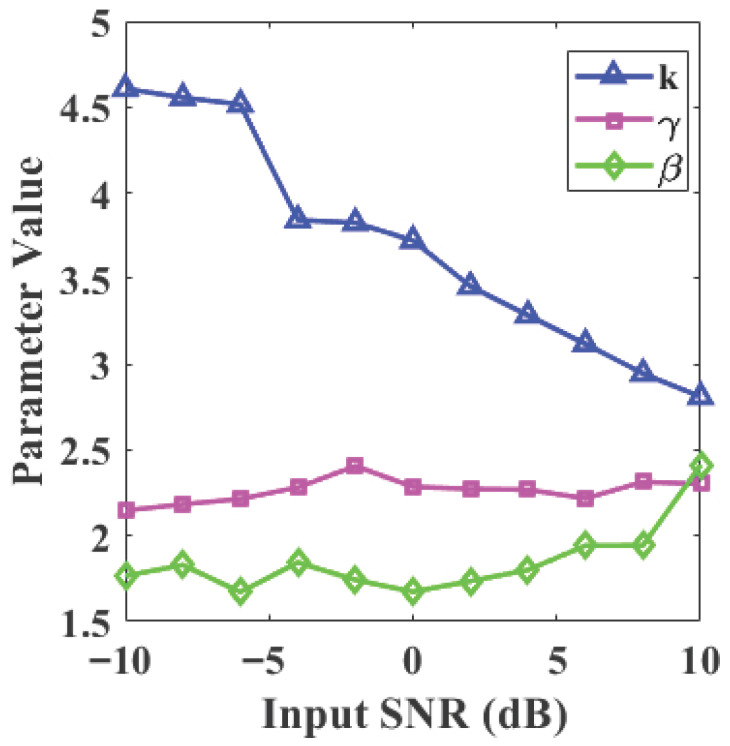
Adaptive parameters.

To further validate the algorithm’s performance across varying noise intensities, this study tests within an input signal-to-noise ratio (SNR) range from −10 dB to 10 dB. The dynamic response of the adaptive parameters *k*, γ, and β is illustrated in Figure 7. As the input SNR increases, the shaping parameter *k* gradually decreases to smooth the threshold transition, while the adjustment factors γ and β respond to the changing noise distribution and signal energy, providing the mathematical basis for the system’s self-regulation.

**Figure 8:**
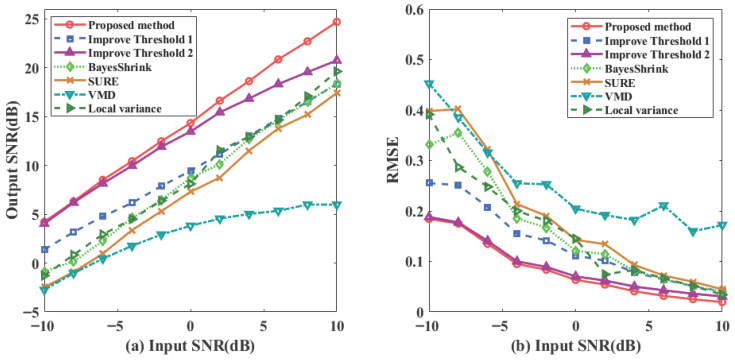
Output metrics for input SNR ranging from −10 to 10. (**a**) SNR. (**b**) RMSE.

The output SNR and RMSE metrics are summarized in Figure 8a,b. The proposed method consistently maintains the highest output SNR and the lowest RMSE across the entire testing range, significantly outperforming the expanded set of baseline methods. The results indicate that in the low input SNR range, the output SNR of the proposed method shows a stable improving trend as the input SNR increases, demonstrating that the proposed mechanism (supported by the parameter curves in Figure 7) effectively enhances noise suppression strength. In contrast, the VMD and local variance methods exhibit substantially lower performance; specifically, VMD maintains an output SNR below 10 dB even at high input SNR levels, reflecting its poor adaptability to composite noise.

In the high input SNR range, the algorithm’s threshold adjustment automatically becomes more conservative, avoiding excessive attenuation of valid signal components and thereby maintaining high waveform fidelity. Concurrently, the RMSE curve remains at the lowest level across the entire SNR range, reflecting superior reconstruction accuracy and stability. Overall, the combination of parameter dynamics in Figure 7 and performance metrics in Figure 8 verifies the self-regulating characteristic of the proposed method—“enhanced suppression under strong noise, minimized intervention under weak noise”—which ensures a balance between denoising performance and signal fidelity across different SNR scenarios.

## 4. Experimental Setup and Analysis

**Figure 9:**
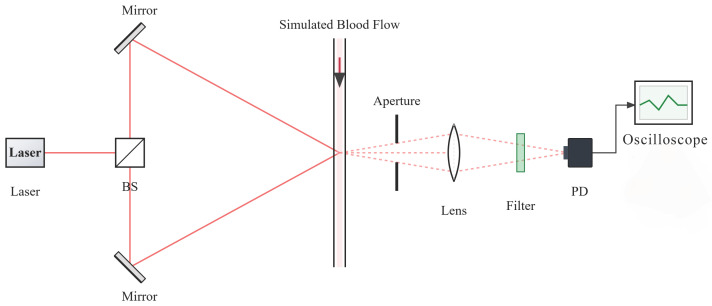
Principle of forward dual-beam dual-scattering blood flow measurement.

This experiment was based on the forward dual-beam, dual-scattering optical measurement principle, which is illustrated in Figure 9. The light source employed was an HNR016P Helium-Neon laser with an operating wavelength of 632.8nm, an output power of ≥1.6mW, and a power stability of ±5%. The laser tube operated in a linearly polarized mode, featuring an extinction ratio of 200:1, a beam divergence of ≤2.0mrad, and a beam diameter of 0.64mm. The detector used was a Thorlabs PDA100A2, with a spectral response range of 320–1100nm. It offered an adjustable signal gain mode, which was set to $0.75\times 10^{4}\;V/A\pm 2\%$ for this experiment. The detector’s noise equivalent power was $3.36\times 10^{-12}\;W/\sqrt{\mathrm{Hz}}$, and its frequency response bandwidth was 800kHz.

**Figure 10:**
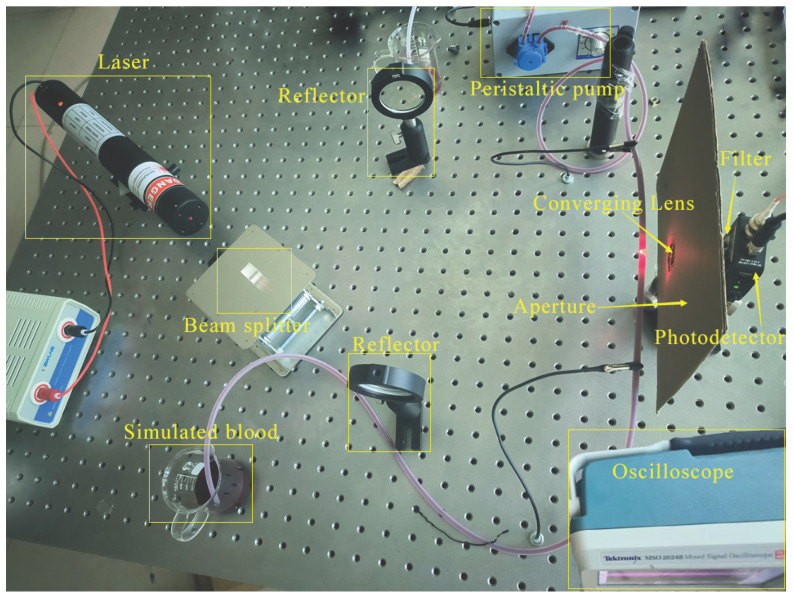
Laser Doppler simulated blood flow detection system.

**Figure 11:**
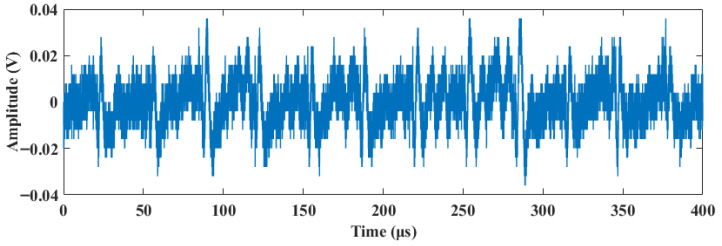
Acquired original signal.

The laser Doppler blood flow detection system constructed in the laboratory is illustrated in Figure 10. The half-angle θ of the included angle between the two laser beams was set to $30^{\circ }$. A 250mL beaker served as the fluid reservoir, with a variable-speed peristaltic pump driving the fluid at a simulated blood flow velocity of 1mm/s. The pump outlet was connected to a silicone tube with an inner diameter of 3mm, acting as a simulated blood vessel channel. To mimic the optical scattering characteristics of real blood, carboxylated monodisperse polystyrene fluorescent microspheres with a diameter of 7 μm were selected as red blood cell substitutes. These were dispersed at a volume concentration of 2.5% in a specialized simulated blood matrix compliant with the medical industry standard YY/T0700. The system’s sampling frequency was 125MHz. The voltage signal output from the photodetector was acquired by an oscilloscope and then imported into MATLAB R2023b software. A waveform segment comprising 50,000 data points extracted from this signal is shown in Figure 11.

**Figure 12:**
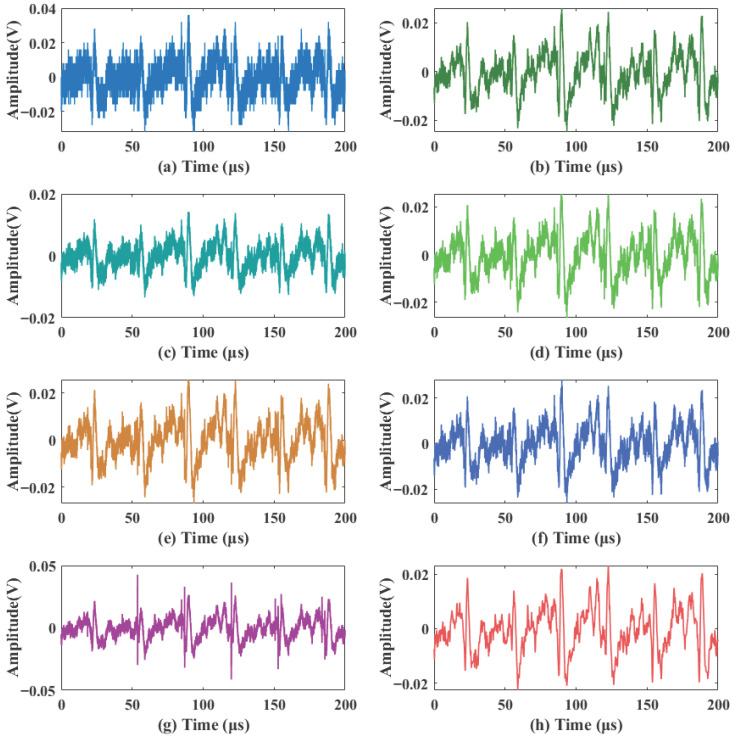
Detail waveforms of the real signals 0–200 μs. (**a**) Acquired original signal; (**b**) local variance; (**c**) VMD; (**d**) BayesShrink; (**e**) SURE; (**f**) Improved Threshold 1; (**g**) Improved Threshold 2; (**h**) proposed method.

**Figure 13:**
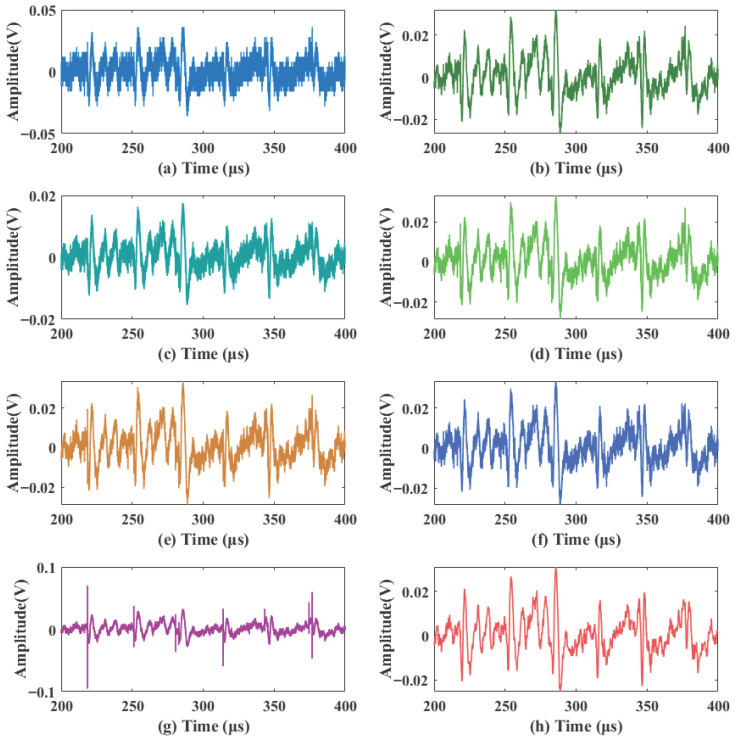
Detail waveforms of the real signals 200–400 μs. (**a**) Acquired original signal; (**b**) local variance; (**c**) VMD; (**d**) BayesShrink; (**e**) SURE; (**f**) Improved Threshold 1; (**g**) Improved Threshold 2; (**h**) proposed method.

The acquired raw signal was severely contaminated by background noise, with a signal-to-noise ratio (SNR) of only −1.04 dB, making the effective features difficult to identify. To validate the performance of the proposed method in a practical scenario, the proposed and various comparison algorithms were applied to the raw signal. To facilitate a more granular observation of the denoising effects and waveform details, the 400 μs signal is divided into two segments: 0–200 μs (shown in Figure 12) and 200–400 μs (shown in Figure 13). The quantitative evaluation results are summarized in Table 3.

As shown in Figure 12 and Figure 13, local variance (b), BayesShrink (d), and Improved Threshold 1 (f) suppress a portion of the thermal noise but leave significant low-amplitude speckle residue. VMD (c) performs poorly across both segments, with persistent high-frequency spikes that severely distort the signal. Notably, while Improved Threshold 2 (g) achieves a relatively stable background in the first 200 μs, it exhibits significant high-amplitude oscillations in the 200–400 μs range (Figure 13g), which could lead to the misidentification of physiological pulses. In contrast, the proposed method (h) maintains a clean, smooth background and consistent baseline morphology across both time segments.

The quantitative results in Table 3 further support these visual observations. The proposed method achieves the highest output SNR (13.86 dB) and the lowest RMSE (0.00258), significantly outperforming the other methods. While the local variance method (b) attains the second-best SNR (12.50 dB), its waveform still contains more micro-fluctuations compared to the proposed strategy. The VMD method (c) yields the lowest performance (SNR = 8.77 dB, RMSE = 0.00542), further proving that the proposed time-domain noise-aware and DWT-based adaptive strategy is more robust against the complex, non-stationary noise typical of practical LDF measurements. This superior performance in preserving signal fidelity while suppressing composite noise demonstrates its strong potential for clinical applications.

## 5. Discussion

This study establishes an adaptive denoising framework for LDF signals, specifically addressing the dual challenges of intense composite noise and signal non-stationarity to enable high-fidelity capture of transient hemodynamic features. While the deterministic physical model used in our experiments assumes single-scattering dominance—which is most suitable for isolated vascular structures, extracorporeal circulation loops, or large vessels—it provides a rigorous “denoising engine” for validating algorithmic performance against a known ground truth. We acknowledge that in transcutaneous diagnostics of skin microvascular beds, multiple random scattering and tissue heterogeneity introduce additional statistical complexity. However, the stochastic nature of the composite noise (e.g., speckle and thermal interference) remains a universal challenge. The proposed SNR-driven strategy, which integrates temporal noise perception and discrete wavelet transform (DWT), effectively addresses this by distinguishing signal-dominant energy from broadband noise, offering a robust foundation that can be extended to more complex, scattering-rich biological environments. Furthermore, as a high-precision velocity tracking tool, this algorithm holds significant potential for non-invasive flow analysis in engineering contexts, such as microfluidics and industrial process control.

## 6. Conclusions

This paper has proposed an adaptive denoising algorithm that integrates temporal noise perception with discrete wavelet transform (DWT) to overcome the inherent limitations of traditional fixed-threshold methods in balancing noise suppression and signal fidelity for LDF signals. Experimental results from both numerical simulations and in vitro phantom tests lead to the following conclusions:

1. The algorithm achieves superior noise suppression, yielding a 15.45 dB output SNR in simulations and 13.86 dB in practical phantom measurements, outperforming traditional wavelet methods (e.g., BayesShrink, SURE) as well as modern benchmarks like VMD and local variance.
2. The SNR-driven self-regulating mechanism eliminates the need for manual hyperparameter tuning, ensuring robust performance across varying noise intensities from −10 dB to 10 dB.
3. The improved smoothing function effectively prevents reconstruction artifacts, thereby preserving the critical morphological features of the blood flow waveform.

Future research will focus on the real-time implementation of the algorithm on embedded hardware platforms, as well as further optimizing its mathematical framework to account for multiple scattering effects in more complex, multi-layered biological tissues and real physiological environments.

## Figures and Tables

**Table 1:** Comparison of decomposition and reconstruction performance for three wavelet bases (db8, sym8, coif5).

Wavelet Type	Mean Squared Error (MSE)	Mean Phase Distortion (rad)
db8	$2.6148\times 10^{-26}$	$1.5876\times 10^{-12}$
sym8	$3.0069\times 10^{-29}$	$1.0012\times 10^{-12}$
coif5	$2.8524\times 10^{-21}$	$2.8337\times 10^{-9}$

**Table 2:** Simulation performance comparison of denoising methods for laser Doppler signals.

Method	SNR (dB)	RMSE
Local variance	9.20	0.12544
VMD	4.20	0.22302
BayesShrink	9.16	0.12601
SURE	8.76	0.14216
Improved Threshold 1	10.34	0.10145
Improved Threshold 2	14.35	0.06391
Proposed method	15.45	0.05634

**Table 3:** Comparison of actual performance of laser Doppler signal denoising methods.

Method	SNR (dB)	RMSE
Local variance	12.50	0.00304
VMD	8.77	0.00542
BayesShrink	11.30	0.00386
SURE	10.98	0.00397
Improved Threshold 1	11.49	0.00363
Improved Threshold 2	9.98	0.00453
Proposed method	13.86	0.00258

## Data Availability

The data presented in this study are available on request from the corresponding author due to privacy.
